# CaGrid Workflow Toolkit: A taverna based workflow tool for cancer grid

**DOI:** 10.1186/1471-2105-11-542

**Published:** 2010-11-02

**Authors:** Wei Tan, Ravi Madduri, Alexandra Nenadic, Stian Soiland-Reyes, Dinanath Sulakhe, Ian Foster, Carole A Goble

**Affiliations:** 1 Computation Institute, University of Chicago and Argonne National Laboratory, Chicago, IL, USA; 2 Mathematics and Computer Science Division, Argonne National Laboratory, Argonne, IL, USA; 3 School of Computer Science, University of Manchester, Manchester, UK

## Abstract

**Background:**

In biological and medical domain, the use of web services made the data and computation functionality accessible in a unified manner, which helped automate the data pipeline that was previously performed manually. Workflow technology is widely used in the orchestration of multiple services to facilitate in-silico research. Cancer Biomedical Informatics Grid (caBIG) is an information network enabling the sharing of cancer research related resources and caGrid is its underlying service-based computation infrastructure. CaBIG requires that services are composed and orchestrated in a given sequence to realize data pipelines, which are often called scientific workflows.

**Results:**

CaGrid selected Taverna as its workflow execution system of choice due to its integration with web service technology and support for a wide range of web services, plug-in architecture to cater for easy integration of third party extensions, etc. The caGrid Workflow Toolkit (or the toolkit for short), an extension to the Taverna workflow system, is designed and implemented to ease building and running caGrid workflows. It provides users with support for various phases in using workflows: service discovery, composition and orchestration, data access, and secure service invocation, which have been identified by the caGrid community as challenging in a multi-institutional and cross-discipline domain.

**Conclusions:**

By extending the Taverna Workbench, caGrid Workflow Toolkit provided a comprehensive solution to compose and coordinate services in caGrid, which would otherwise remain isolated and disconnected from each other. Using it users can access more than 140 services and are offered with a rich set of features including discovery of data and analytical services, query and transfer of data, security protections for service invocations, state management in service interactions, and sharing of workflows, experiences and best practices. The proposed solution is general enough to be applicable and reusable within other service-computing infrastructures that leverage similar technology stack.

## Background

For years, web-based systems have provided biological and medical scientists access to various data and computation resources to facilitate their scientific exploration. To achieve a fully functional data pipeline, scientists used to switch among browsers, copy from one web page, convert the obtained data and paste it to another one. The emergence of web services made their functionality accessible by computer programs which helped automate the data pipeline that was previously performed manually. Today, the number of available web services has grown significantly. BioCatalogue [1], a curated catalogue of life science web Services, has collected more than 1400 services from over 100 providers, and a paper [2] from 2006 reported 3000 publicly available services in molecular biology.

Cancer Biomedical Informatics Grid (caBIG) [3], sponsored by the US National Cancer Institute (NCI), is an information network enabling cancer researchers and physicians to share data and knowledge, and thus accelerate the discovery of new cancer treatment methods. Cancer Grid (caGrid) [4] is the underlying infrastructure of caBIG, and is built on the Globus toolkit Grid middleware [5]. CaGrid consists of web services as virtualized access points of data and analytical resources related to cancer detection, diagnosis, treatment and prevention. As of May 2010, caGrid hosts more than 140 data and analytical services. Despite the diversity of cancer research related resources, caGrid organizes them into two categories: data resources are exposed as *data services*; analysis applications are exposed as *analytical services*. Typical data services include human biospecimens, entrez genes, microarray, etc; typical analytical services include gene alignment, clustering, classification, principle component analysis, etc. Most of caGrid services are accessible by all researchers without any security requirement. Some of them limit their access to certain caGrid users, but researchers can obtain a caGrid credential easily and contact service owners for specific access permit.

Due to diverse purposes and approaches of scientific investigations performed by researchers, few web services alone can fulfill the requirement of an in-silico experiment. Instead, it is often required that services are composed and orchestrated in a given sequence to realize data pipelines, which are often called *scientific workflows*.

Many scientific workflow systems, such as Kepler [6], Triana [7], Trident [8] and Taverna [2], and parallel scripting systems, such as Swift [9] and Pegasus [10], are available today to aid the execution of workflows. All these systems support the composition of local or remote executable components and execute them in a predefined sequence.

caGrid project selected Taverna as its workflow execution system of choice due to its integration with web service technology and support for a wide range of web services, plug-in architecture to cater for easy integration of third party extensions, and a broad user base within the bioinformatics/biomedicine community [11]. The caGrid Workflow Toolkit, an extension to the Taverna workflow system, is designed and implemented to ease building and running caGrid workflows. It provides users with support for various phases in using workflows: service discovery, composition and orchestration, data access, and secure service invocation, which have been identified by the caGrid community as challenging in a multi-institutional and cross-discipline domain.

1. **Service discovery **-where to find services that are relevant to the scientific investigation of the user.

2. **Data access **- what kind of data (data types) can be obtained from a given service and how to transfer data from and to it.

3. **Service interaction **- how to invoke services and maintain the session information in multi-steps interactions.

4. **Security enforcement **- how to enforce authentication and authorization in service invocations and privacy and integrity in data transfers.

5. **Knowledge sharing **- how to share workflows with the community, how to find out what other researches in the field are doing and to leverage the best practice from them.

We have followed two principles in the design and development of the toolkit. Firstly, instead of reinventing the wheel, we have adopted the software tools which are widely used by the life science community, namely Taverna and myExperiment [12]. We have provided added value to these tools by offering more advanced features in form of plug-ins that make caGrid infrastructure accessible from Taverna. Secondly, we have worked closely with scientists from caBIG to fulfill their needs, while making our tool applicable to a broader user community that embraces a similar service infrastructure.

## Implementation

**Figure 1:**
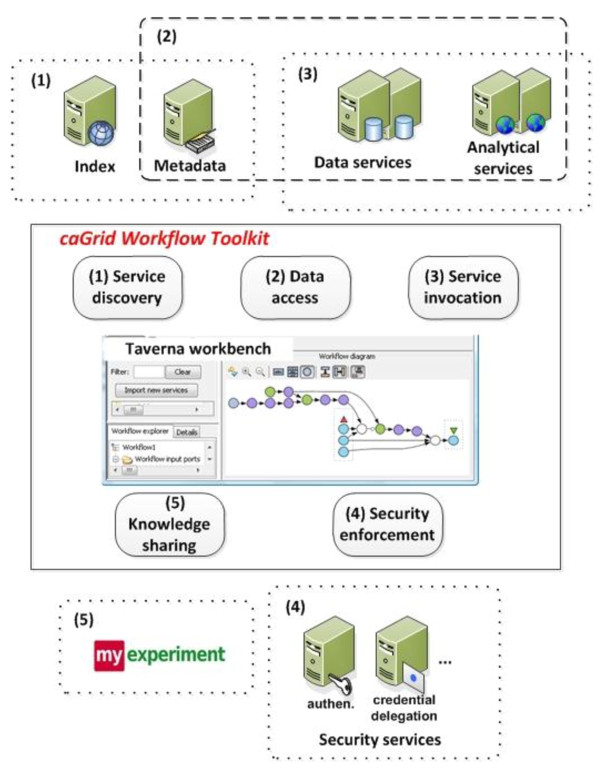
**Architecture of caGrid workflow toolkit**. The solid rectangle comprises the components of the toolkit, and each component is numbered to illustrate their interactions with the environment.

Figure 1 shows the architecture of the caGrid Workflow Toolkit. The solid rectangle in the middle consists of the five components which are extensions to Taverna. The components in dashed rectangles are modules in caGrid infrastructure and myExperiment. They are numbered in accordance with the toolkit's components to prescribe the interactions in between. *Service discovery *component locates caGrid services by querying the *Index Service *(which is the centralized service registry of caGrid) and caGrid *Metadata Service *(which defines the data types used by all caGrid services). *Data access *component provides: 1) a GUI to build query clauses against data service, and 2) a data transfer utility to move files from and to services. *Service invocation *component enables the stateful interactions with services. *Security enforcement *component ensures the privacy, integrity, authentication and authorization in services invocation and data transfer. *Knowledge sharing *component shares workflows and best practices using myExperiment.

Taverna Workbench provides an extensible framework to interact with various executables, in our case, caGrid services. The caGrid Workflow Toolkit containing caGrid-specific extensions can be downloaded and installed in Taverna Workbench by pointing its *Plug-in Manager *to http://software.cagrid.org/taverna2/. Please note that the five logical components in Figure 1 correspond to only four physical plug-ins, i.e., *cagrid-activity*, *cql-builder*, *cagrid-transfer-activity *and *cds-activity*. This is because the five components on the diagram give a more abstract impression of the features the toolkit offers. However, in the actual software implementation we needed to comply with the Taverna plug-in infrastructure that resulted in spreading the logical functionality to four plug-ins. The correspondence between the logical components and the actual Taverna plug-ins is explained in Table 1.

**Table 1:** Correspondence between logical components and Taverna plug-ins

Components	Taverna Plug-ins	Notes
Service discovery	cagrid-activity	cagrid-activity plug-in contains a service discovery tool

Data access	cql-builder	A GUI to build CQL clauses
	
	cagrid-transfer-activity	An activity to transfer files between services and clients

Service interaction	cagrid-activity	cagrid-activity plug-in takes care of stateful service invocation

Security enforcement	cagrid-activity	cagrid-activity plug-in takes care of secured service invocation
	
	cds-activity	An activity to delegate credential

Knowledge sharing	N/A	This component simply uses myExperiment website.

### Service discovery

In a typical service-oriented infrastructure like caGrid, the address of a service of interest is not usually known to end users. This makes the task of locating appropriate services a challenge for the user, given the fact that caGrid now comprises more than 140 services storing different data or providing varied analysis capabilities, which are deployed at geographically distributed institutions.

On top of the Globus Index Service [13], caGrid provides the mechanism to discover services of interest by querying a live service registry. All caGrid services are required to publish the metadata that describes their functionality using the WSRF (Web Service Resource Framework) [14], a family of specifications for web services to expose state and property information. This information is aggregated in the registry Index Service and used to find out information about the currently running services and their current WSDL addresses. Clients can then query this aggregated information using standard WSRF operations.

The services' descriptive metadata includes service name, WSDL, hosting research center, operations with associated data types, and the semantic annotation on the aforementioned metadata. Service discovery component locates caGrid services by querying the Index Service. It provides three types of querying methods leveraging caGrid's metadata and indexing infrastructure:

• String based querying performs free text searching in service descriptions. For example, one can search for services whose descriptions contain string '*array'*.

• Property based querying performs search towards pre-defined service properties. For example, to locate services *hosted *by *NCICB *(the NCI Center for Bioinformatics), or whose *name *is *CaArraySvc*, etc.

• Semantic based querying. caGrid uses an ontology called NCI Enterprise Vocabulary Services (EVS) [15] to annotate services and their associated data. A vocabulary item in EVS is called *Concept *and the *Concept Code *is used to uniquely identify it. Semantic based approach allows users to locate services which are annotated with some concept code (for example, *C44282 *representing concept *Microarray*).

### Data access

caGrid Data Service [16] is used to share cancer research data. CaGrid data services implement an object-oriented virtualization on top of the backend data source. Based on this virtualization, data items can be searched for by their object classes, properties, and association relations. caGrid also defines a XML based object oriented query language called caGrid Query Language (CQL) [17] for querying purpose. Data access component provides a GUI to build CQL queries against data services, and users can browse the data object model graph and build a CQL clause easily without knowing the syntax of it.

Another feature offered by the data access component is the data transfer tool called caGrid transfer. It leverages the caGrid transfer utility [18] to move files between services and clients using HTTP protocol, without embedding them in SOAP messages. In our practice, we found this is more efficient since it avoids data serialization or deserialization and saves a lot of memory on both client and server side.

### Service invocation

CaGrid services use WSRF extension to enable stateful communications with clients. *Service invocation *component implements WSRF specification so that it can interact with services in a stateful manner. This feature is extremely useful in a multi-step interaction with a service, which is quite common in scientific applications. For example, a scientist submits a data set to a caGrid service to run a computation-intensive task. Since the computation usually takes some time in the backend system, result cannot be returned in a synchronous manner. In this case the service chooses to synchronously return an EPR (End Point Reference) which identifies the state of the service interaction. At a later time, the scientist uses the EPR as a handler to query the status of the task that he submitted earlier, and obtain the result when it is ready. The WSRF implementation on the server side uses the EPR to identify the instance of the service and return the specific result data appropriately. The issuance and management of an EPR is handled by the Globus toolkit and the service invocation component, and is transparent to users.

### Security enforcement

Security is an important aspect in biomedical applications. Scientists want to ensure the privacy, integrity, authentication and authorization in the sharing of data and computation resources in a multi-institutional environment. For example, scientists may constrain the access to their data to certain organizations or groups of users (authentication and authorization); they may want to access their data in an encrypted way so no other people can intercept the content (privacy); they may also want an assurance that the data the recipient gets is exactly the same as sent by the sender (integrity). All these issues have been addressed by the Grid Security Infrastructure (GSI) [19] in the Globus toolkit. GSI leverages Public Key Infrastructure (PKI) and X.509 certificates [20] to achieve these security requirements.

caGrid devised the Grid Authentication and Authorization with Reliably Distributed Services (GAARDS) [21] as an extension to the GSI to provide services and tools for the administration and enforcement of security policies in caGrid. *Security enforcement *component (component (4) in Figure 1) allows users to log in to caGrid, obtain a grid credential from the Authentication Service, store it locally and use it for subsequent service invocations for the lifetime of the credential (effectively achieving single sign-on). In addition to this single-sign-on feature, the security enforcement component also allows credential delegation so that a service can act on behalf of the user. For example, the Federated Query Processor (FQP) service can use a delegated credential from a user, query multiple data services on the user's behalf, aggregate the results and forward them back to the user. More comprehensive explanation and an example are given in the results section.

### Knowledge sharing

myExperiment [12] is a sister project of Taverna and a web-based collaborative platform for sharing workflows and related research objects such as data items, papers, software bundles, etc. *Knowledge sharing *component simply uses myExperiment website to publish the workflows [22] built and used by caGrid community. These workflows contain detailed descriptions of what the workflows are set to achieve and instructions on how to use them (e.g. what data to use as input). They also embed knowledge on how to use individual services as well as how to orchestrate multiple services into a full-fledged data pipeline. These workflows range from simple tasks such as querying microarray data and retrieving medical images, to full-fledged routines such as federated query over multiple data sources and lymphoma type prediction (see Scenario 4 in the Results section). For space limit we do not iterate over all the workflows and users are encouraged to visit the link given in [22] to find the ones they are interested in. These workflows are modelled in a graphical way and organized into meaningful modules, so that they can easily be reused out of the box, or be modified and repurposed.

## Results

**Figure 2:**
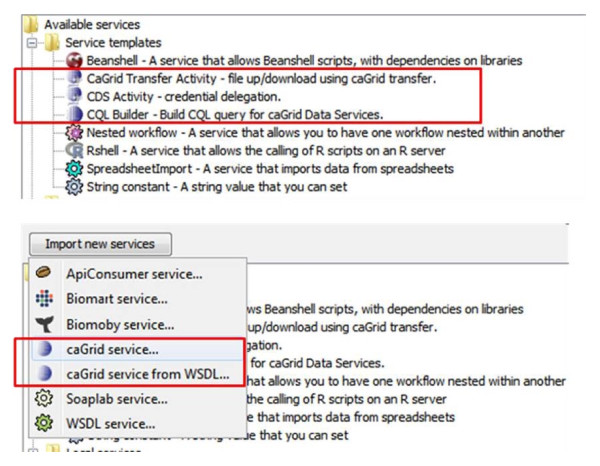
**All four plug-ins in Taverna service panel**. The caGrid workflow toolkit currently contains four plug-ins, i.e., cagrid-activity (*caGrid service*... and *caGrid service from WSDL...*) for service discovery, invocation and security enforcement; cql-builder (*CQL Builder*) for visualized construction of CQL clause to query data services; cagrid-transfer-activity (*CaGrid Transfer Activity*) for file transfers between clients and services; cds-activity (*CDS Activity*) for credential delegation.

Once caGrid Workflow Toolkit is installed in the Taverna Workbench, the four plug-ins show up in Taverna's Service Panel (see Figure 2): cagrid-activity plug-in (see *caGrid service *and *caGrid service from WSDL *in Figure 2) for service discovery, invocation and security; cql-builder plug-in (*CQL Builder*) for visualized construction of CQL against data services; cagrid-transfer-activity plug-in (*CaGrid Transfer Activity*) for file transfers between clients and services; cds-activity plug-in (*CDS Activity*) for credential delegation. These plug-ins can be utilized from workflows to access caGrid functionality.

In this section, we show the usage of these plug-ins through several typical application scenarios. A more complete list of functions can be found from the user manual and more example workflows from myExperiment [22]. For a more complete reference on how to use other types of services shown in Figure 1, please refer to [2,23,24].

### Scenario 1: service discovery

**Figure 3:**
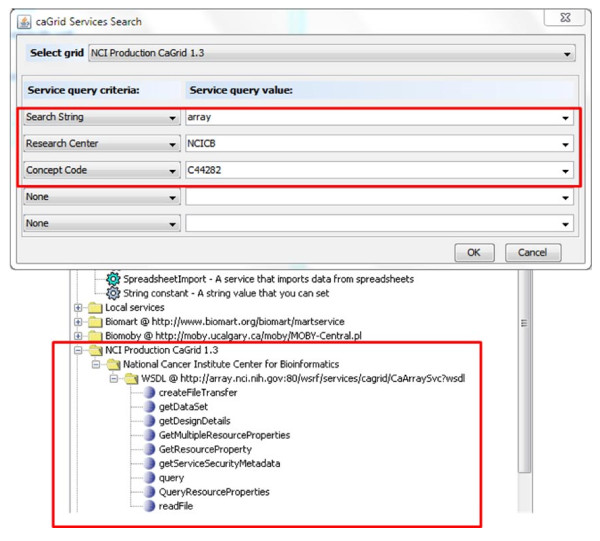
**Service discovery GUI and the result**. Search services whose description contains *array*, hosted by *NCICB*, and annotated with concept code *C44282*.

As shown in Figure 2, there are two ways to add a caGrid service into a Taverna workflow. In the case WSDL URL of a caGrid service is known, it can be directly added to Taverna's Service Panel. In a more general case, when users do not know which service(s) to use, they first need to use the service discovery component. Figure 3 illustrates a service discovery combining the string, property and semantic based approaches. In this case, we look for services whose description contains *array*, are hosted by *NCICB*, and are annotated with concept code *C44282*. Figure 3 shows the search dialog (the upper part) and the result service with operations (the lower part). The discovery result is the NCICB hosted caArray service, to be explained in more detail in scenario 2 next.

### Scenario 2: access of caArray data using cql-builder and cagrid-transfer

caArray [25,26] is an open-source, web and programmatically accessible microarray data management system developed by caBIG. Since it acquires, disseminates and aggregates a large volume of cancer related array data, cancer researchers in caBIG frequently start their in-silico investigation by querying and retrieving data from caArray, and subsequently analyze them using tools and services on and off the caGrid.

**Figure 4:**
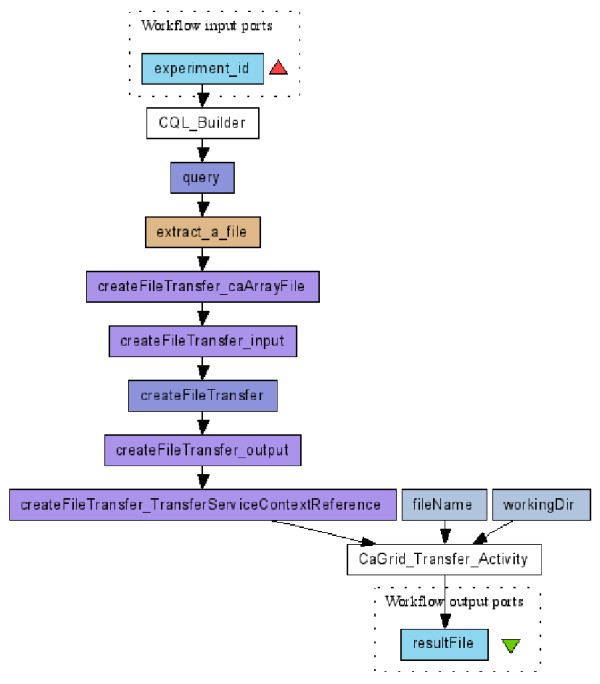
**Query caArray data service and retrieve files **[33]. *CQL_Builder *provides a GUI to build a complex CQL clause querying caArray files. *CaGrid_Transfer_Activity *downloads files to a local directory using caGrid transfer utility.

**Figure 5:**
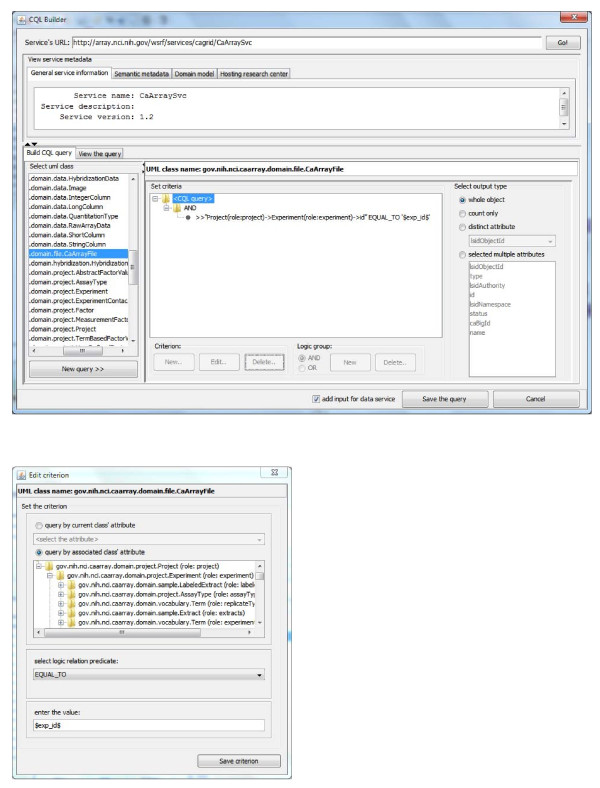
**CQL builder to construct CQL querying clause to caArray service**. *CQL Builder *dialog provides a GUI to build a complex CQL clause querying data services. The *Edit criterion *dialog is used to build querying criteria in CQL.

Figure 4 shows a workflow that queries all the files related to a microarray experiment, and selects and downloads some of them. The input of the workflow is the identifier of the microarray experiment (*experiment_id*) of interest. Within the CQL builder (*CQL_Builder*) a complex CQL clause is built, querying all the caArray file objects associated with this experiment (see the CQL builder GUI and the criteria editing dialog in Figure 5). A user can then pick up one or several caArray files (*extract_a_file*), create a download session (*createFileTransfer*) and use the cagrid-transfer-activity plug-in (*CaGrid_Transfer_Activity*) to download files to a local directory. The output is the name of the downloaded file (*resultFile*). From now on, we only mention the key activities/services in a workflow, and those that are not mentioned may be local activities doing data transformation, xml manipulation, etc.

In the CQL builder GUI (the upper dialog of Figure 5), users can choose which service to query by giving the service's URL, and then its object-oriented data model is populated and ready to be selected. Users can select one of them (*gov.nih.nci.caarray .domain.file.CaArrayFile*) and edit the criteria clause (the GUI to edit query criteria is shown in the lower part of Figure 5).

### Scenario 3: secured query and credential delegation

The caGrid Federated Query Processor (FQP) service provides a mechanism to perform distributed queries over multiple data services and result aggregation. FQP is a secure service so that a user who invokes it needs to use his X.509 certificate to encrypt or sign the invocation message. However, it is not sufficient that the FQP authenticates the invoker, since the FQP subsequently needs to query multiple data services on behalf of the invoker. Therefore the FQP must be supplied with the invoker's credential so that those data services can give FQP the same privileges they would give to the original invoker.

GSI introduces X.509 proxy certificates [20] that allow a user (the service invoker in our case) to assign dynamically a new X.509 identity to an entity (the FQP in our case) and then delegate a subset of his rights to that entity. Users create a proxy certificate by issuing a new X.509 certificate signed using their own credentials instead of involving a CA. In caGrid, the Credential Delegation Service (CDS) is a WSRF-compliant Grid service that enables users/services (delegator) to delegate their Grid credentials to other users/services (delegatee) such that the delegatee(s) may act on the delegator's behalf.

**Figure 6:**
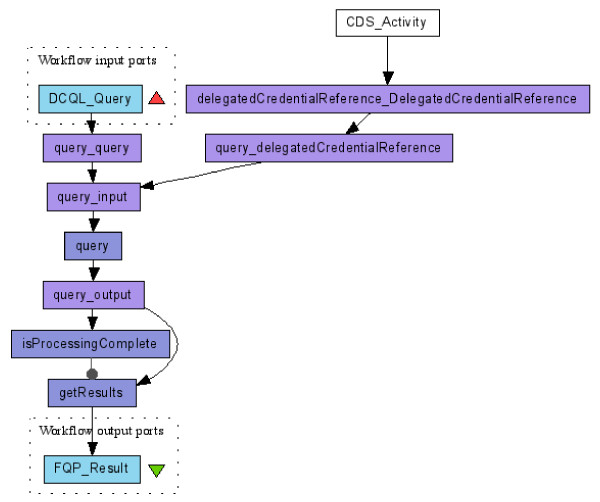
**Invoke caGrid FQP securely and use credential delegation **[34]. *CDS_Activity *issues an EPR of the delegated credential. FQP uses this EPR to fetch the actual delegated credential also from CDS and uses it to invoke multiple data services (the *query *activity) on behalf of the invoker.

In Figure 6, the FQP service (the *query *activity) is configured as a secure service and the user of this workflow needs to provide his certificate to invoke it along with a CQL clause (workflow input *DCQL_Query*). Besides that, the invoker needs to ask CDS to issue a delegated credential and return the EPR of it (*CDS_Activity*). FQP uses this EPR to fetch the actual delegated credential also from CDS and uses it to invoke multiple data services on behalf of the invoker, aggregate and return the results (the *getResult *activity) to the invoker.

### Scenario 4: lymphoma type prediction as a complex case

Here we describe a real-life workflow built for caBIG users to illustrate a fairly complex scenario. The workflow enables cancer diagnosis based on microarray analysis [27].

**Figure 7:**
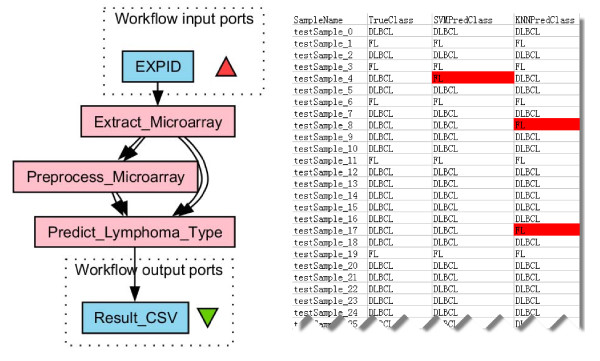
**lymphoma type prediction workflow and the result**. Microarray data is extracted from caArray, preprocessed and used to learn a model for lymphoma type prediction. Result is a csv file describing the actual lymphoma type of each tumor sample and the prediction results using SVM and KNN algorithms, respectively.

**Figure 8:**
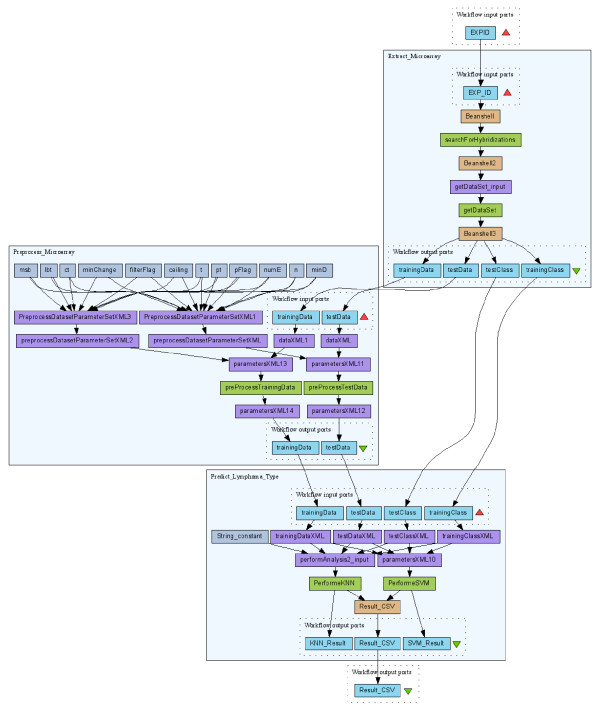
**The complete lymphoma type prediction workflow **[35].

As shown in Figure 7, the workflow starts with the extraction of hybridization data from a given experiment in the aforementioned caArray database (nested workflow *Extract_Microarray*). These hybridizations are from tumor samples that belong to two different lymphoma types, i.e., diffuse large B-cell lymphoma (DLBCL) and follicular lymphoma (FL). Next, the hybridization data are pre-processed (nested workflow *Preprocess_Microarray*) and then used to learn a classification model using two machine learning methods, i.e., Support Vector Machine (SVM) and K-Nearest Neighbor (KNN). This model is used for lymphoma type prediction when an unknown sample comes in (nested workflow *Predict_Lymphoma_Type*). The type prediction result is shown in the right part of Figure 7. *SampleName *represents different tumor samples; *TrueClass *is the lymphoma type obtained by manual investigation (and is considered to be accurate); *SVMPredClass *and *KNNPredClass *represent the types predicted by SVM and KNN, respectively. Prediction errors are highlighted. While Figure 7 shows the skeleton of the lymphoma workflow by condensing the nested workflows, Figure 8 gives a detailed view with nested workflows expanded.

## Discussion

Several other Taverna extensions for specific uses exist, such as CDK-Taverna [28] and an R-extension [29]. CDK-Taverna is an extension to access a cheminformatics library called CDK, and the R-extension allows users to submit a statistical calculation job to a remote R server. The caGrid extension of Taverna is implemented from the perspective of service computing, enabling users to access a broad range of remote services in a more standard, secure and scalable manner. In this way scientists can greatly improve their productivity by accessing powerful information provision tools and automate their data analysis, without knowledge of, or control over, the internal workings of those tools. In addition to caGrid services, we have successfully applied the caGrid Workflow Toolkit in other projects which use similar services technology stack, for example, the CardioVascular Research Grid (CVRG) [30]. This effort is part of the *Service-oriented Science *mission [31].

New challenges rise in the caBIG community when more users rely on the caGrid Workflow Toolkit to document and execute their in-silico experiments. While the current toolkit offers day-to-day functions to search, compose, orchestrate services and share workflows, it does not address much of knowledge transfer and reuse. A framework called CaaS (Composition-as-a-Service) [32] is positioned to overcome the isolated nature of current service composition approaches in which knowledge can neither accumulate nor be shared among people who do similar research. In short, CaaS is a recommendation framework that provides composition recommendations to stakeholders and collects feedback from them. It leverages cutting edge technologies like social network analysis, web 2.0, recommendation systems, etc.

## Conclusions

In biomedicine and bioinformatics, service computing infrastructure now plays a key role in the integration of various data and computational resources in a uniformed manner. Workflow technology is widely used in the orchestration of multiple services to facilitate in-silico research. By extending the Taverna Workbench, caGrid Workflow Toolkit provided a comprehensive solution to compose and coordinate services in caGrid, which would otherwise remain isolated and disconnected from each other. Using it users can access more than 140 services and are offered with a rich set of features including discovery of data and analytical services, query and transfer of data, security protections for service invocations, state management in service interactions, and sharing of workflows, experiences and best practices. Although we currently focus on application domains such as cancer (caBIG) and cardiovascular (CVRG), the proposed solution does not limit itself to any specific application and general enough to be applicable and reusable within other service-computing infrastructures.

## Availability and requirements

* Project name: caGrid Workflow Toolkit

* Project home page: http://wiki.cagrid.org/display/workflow/

* Operating system(s): Platform independent

* Programming language: Java

* Other requirements: Java 1.6.0 or higher, http://java.sun.com/. Taverna 2.1.2,

http://www.taverna.org.uk/download/taverna-2-1/

* License: caBIG^® ^Open Source Software License caGrid 1.3,

http://cagrid.org/display/downloads/caGrid+1.3+License

* Any restrictions to use by non-academics: none

## Authors' contributions

RM and IF conceived the project and led its design and coordination. WT was responsible for writing the manuscript. WT developed the caGrid Workflow Toolkit with the help from DS, SS-R and AN, and composed the caGrid workflows uploaded to myExperiment. SS-R and AN are developers on the Taverna and myGrid projects which are led by CAG. SS-R and AN also developed the initial version of cagrid-activity plug-in. All of the authors have read and approved the final manuscript.
